# Comparison of Vaccine Effectiveness Against the Omicron (B.1.1.529) Variant in Hemodialysis Patients

**DOI:** 10.1016/j.ekir.2022.04.005

**Published:** 2022-04-13

**Authors:** Katrina J. Spensley, Sarah Gleeson, Paul Martin, Tina Thomson, Candice L. Clarke, Graham Pickard, David Thomas, Stephen P. McAdoo, Paul Randell, Peter Kelleher, Rachna Bedi, Liz Lightstone, Maria Prendecki, Michelle Willicombe

**Affiliations:** 1 Centre for Inflammatory Disease, Department of Immunology and Inflammation, Imperial College London, London, UK; 2 Imperial College Renal and Transplant Center, Imperial College Healthcare NHS Trust, Hammersmith Hospital, London, UK; 3 Department of Infection and Immunity Sciences Northwest London Pathology NHS Trust, Charing Cross Hospital, London, UK; 4 Department of Infectious Diseases, Imperial College London, London, UK

**Keywords:** COVID-19, SARS-CoV-2, haemodialysis

## Introduction

Since its first detection in November 2021, the SARS-CoV-2 B.1.1.529 (Omicron) variant has rapidly become the dominant variant worldwide. Increased transmissibility and its ability to evade both vaccine- and infection-induced neutralizing antibodies were of significant global concern and the impetus for the United Kingdom to expedite its booster vaccination program.^1^ *In vitro* and animal models suggested that although demonstrating the unfavorable characteristics described, the pathogenicity of the Omicron variant was reduced.^2^^,^^3^ The emerging real-world data seem to mirror the laboratory findings, with data revealing a reduction in vaccine effectiveness (VE) against infection with Omicron but enhanced efficacy against severe infection requiring hospitalization.^4^

For clinically vulnerable populations, a less pathogenic variant may still have significant impact on morbidity and mortality. People with kidney failure receiving in-center hemodialysis are one such patient group. Patients receiving in-center hemodialysis have attenuated responses to SARS-CoV-2 vaccines, with recent data suggesting patients who have received 3 doses of a heterologous vaccination regimen may have inadequate neutralizing ability against Omicron, leaving them at significant risk of infection.^5^^,^^6^ Here, we assess the clinical impact of Omicron infection, and VE, in an in-center hemodialysis population followed up within a prospective longitudinal surveillance study at the Imperial College London (HRA REC reference: 20/WA/0123).

## Results

A total of 1121 in-center hemodialysis patients were included in the analysis; all patients underwent weekly screening for SARS-CoV-2 infection via reverse transcriptase-polymerase chain reaction (RT-PCR) testing. Screening for infection via weekly (RT-PCR) testing and 3-monthly serologic assessment started before the vaccine rollout in 2020 (Supplementary Methods). Between December 1, 2021, and January 16, 2022, SARS-CoV-2 infection was diagnosed in 156 of 1121 patients (13.9%), equating to an infection rate of 3.1 per 1000 patient days (Supplementary Figure S1). Infection with Omicron was diagnosed in 145 of 156 cases (92.9%); 54 (37.2%) by genotyping, 80 (55.2%) by S-gene target failure, and 11 (7.6%) were classified as “probable cases.” A summary of patient characteristics of Omicron-infected and noninfected patients may be found in the Supplementary Table S1. A total of 11 additional cases were attributed to infection by the Delta variant, with confirmation via sequencing in 9 cases (81.2%). Of 1110 remaining patients, 71 (6.4%) were unvaccinated, 293 (26.4%) were partially vaccinated, and 747 (67.3%) had received 3 doses of vaccine.

**Figure 1:**
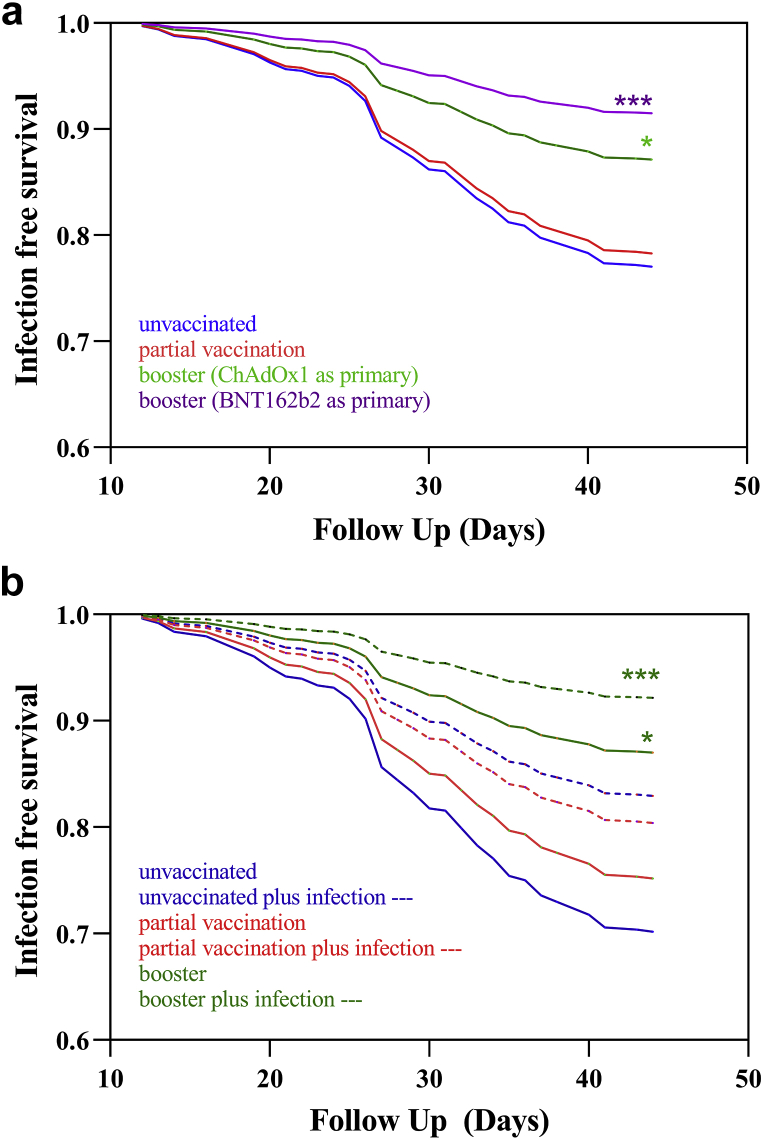
(a) Infection event rate by vaccination status and primary vaccine type. No difference in infection events was found between patients who were unvaccinated and partially vaccinated, HR 0.94 (0.54–1.72), *P* = 0.83. Patients who had received a booster dose had less infective events than unvaccinated patients whether they had received primary with ChAdOx1, HR 0.53 (0.30–0.98), *P* = 0.034, or BNT162b2, HR 0.34 (0.19–0.64), *P=* 0.0005. (b) Survival curve by vaccination status and prior infection. There was no difference in infection events in unvaccinated patients compared with unvaccinated patients with prior infection, HR 0.53 (0.18–1.47), *P* = 0.23, partial vaccination in infection-naive patients, HR 0.81 (0.39–1.82), *P* = 0.58, or patients with partial vaccination who were infection naive, HR 0.62 (0.30–1.38), *P* = 0.20. Patients who were boosted with or without prior infection experienced less Omicron infection episodes, HR 0.23 (0.11–0.52), *P* = 0.0001 and HR 0.39 (0.20–0.86), *P* = 0.01, respectively. HR, hazard ratio. ∗, *P* = <0.05*.* ∗∗∗, = *P* < 0.01*.*

A summary of patient characteristics for those receiving BNT126b2 and ChAdOx1 primary vaccination course may be found in Supplementary Table S2. Unadjusted and adjusted VE against Omicron infection was 58% (23%–75%) (*P* = 0.002) and 50% (8%–71%) (*P* = 0.018), respectively, in patients who had received a booster vaccine, whereas no efficacy was found in patients who had only been partially vaccinated (Supplementary Table S3). Analyzing VE in the 747 patients who had been boosted, significant effectiveness was found in both patients who received ChAdOx1, VE of 47 (2–70)% (*P* = 0.034), and BNT162b2, VE of 66 (36–81)% (*P* = 0.0005), as their first 2 doses (Figure 1a, Supplementary Table S4).

There were 579 of 1110 (52.2%) patients who had evidence of prior infection at the start of follow-up, 63 of 145 (43.4%) patients who subsequently were diagnosed with Omicron infection, and 516 of 965 (53.5%) patients who remained infection free. Although reinfections were found, prior infection reduced the unadjusted and adjusted likelihood of Omicron infection, hazard ratio 0.69 (0.50–0.96), *P* = 0.029 and hazard ratio 0.63 (0.45–0.87), *P* = 0.0059, respectively (Supplementary Table S3). Analyzing prior infection by vaccine status, prior infection alone, hazard ratio 0.53 (0.18–1.47), *P* = 0.23, or prior infection with partial vaccination, hazard ratio 0.62 (0.30–1.38), *P* = 0.20, did not reduce the likelihood of infection. For patients who were boosted, a VE of 61% (14%–80%), *P* = 0.01, was found in those without prior infection and 77% (48%–89%), *P* = 0.0001, in those with prior infection (Figure 1b and Supplementary Table S3).

With a median follow-up of 25 (interquartile range: 19–28) days postdiagnosis, 4 of 145 patients (2.8%) died within 28 days of infection. Of 145, patients, 4 (2.8%) acquired infection via nosocomial transmission and 2 of these patients died. Of the remaining 141 patients who were diagnosed within the outpatient setting, 12 (8.5%) were hospitalized at a median of 7 (interquartile range: 2.5–9.5) days postdiagnosis. Of 128 patients, 76 patients (59.4%) who remained outpatients received no directed therapy, compared with 5 of 17 patients (29.4%) who were hospitalized either at the time or after diagnosis (Supplementary Table S5).

## Discussion

We have revealed that 2 doses of a SARS-CoV-2 vaccine fail to provide protection against Omicron infection. VE returns after a booster, irrespective of whether the priming was achieved with BNT162b2 or ChAdOx1. Although reinfections were common, prior infection remained clinically important in reducing the likelihood of infection, which supports immunogenicity data on breadth and durability of immune responses after infection and vaccination.^7^ This may also explain why vaccination failed to demonstrate effectiveness against hospital admission, but prior infection did (Supplementary Table S6), although this may also represent a selection bias of less comorbid patients surviving previous, more pathogenic variants.

Although immunogenicity data have revealed relatively good immunologic responses to SARS-CoV-2 vaccines, particularly mRNA-based vaccines, in patients with kidney failure on hemodialysis, responses are still weaker compared with healthy controls.^6^^,^^8^ Recently, 2 *in vitro* studies have also revealed the necessity of a booster dose in dialysis patients; the first revealed high levels of seropositivity against the Delta and Omicron variants measured by spike glycoprotein cross-reactivity, whereas the second demonstrated enhanced neutralization against Omicron after the booster dose.^5^^,^^9^ The former study suggests no difference between those patients primed with ChAdOx1 compared with a mRNA vaccine, whereas the latter suggests that a significant proportion of patients primed with ChAdOx1 have undetectable neutralizing antibodies post-third dose.^5^^,^^9^ We found no significant difference in clinical outcomes with different primary vaccine courses in this analysis, but this requires further monitoring.

Overall mortality rates in patients with breakthrough infection were much lower than reported in previous waves.S1 In addition to vaccination, another layer of protection against severe disease in this and other vulnerable populations was the introduction of treatments for SARS-CoV-2 in nonhospitalized patients in December 2021 in the United Kingdom. Both agents available, the antiviral molnupiravir and neutralizing antibody, sotrovimab, have been found to reduce disease progression in phase 3 clinical trials.S2,S3 In a common predicament, however, patients with kidney failure were excluded from these trials, and the effectiveness (against Omicron) and potential safety profile of these medications in hemodialysis patients are therefore unknown. Although no safety concerns were reported in our patient cohort, limited inference can be made on the use of molnupiravir because of numbers treated, and further assessment must be made in these patients.

In conclusion, within a hemodialysis population, 3 doses of a SARS-CoV-2 vaccine are required for clinical protection against SARS-CoV-2 Omicron infection. The Omicron variant seems to result in less severe disease compared with other variants in this preliminary analysis. Despite some reassurance from these data, this multimorbid population requires close surveillance, with rapid adaption of vaccine regimens and available treatments, as and if, evidence changes.

## Disclosure

All the authors declared no competing interests.

## Supplementary Material

**Supplementary Methods.**

**Figure S1.** Flow diagram of study cohort.

**Table S1.** Clinical characteristics associated with Omicron infection in haemodialysis patients.

**Table S2.** Clinical characteristics of patients receiving BNT1262b2 compared with ChAdOx1.

**Table S3.** Clinical characteristics associated with Omicron infection.

**Table S4.** Risk of SARS-CoV-2 infection by vaccination status and prior infection.

**Table S5.** Treatments received by care setting.

**Table S6.** Vaccine effectiveness against hospitalisation by vaccination status and prior infection.

**Supplementary References.**
