# Supplementary material for: Comparison of Vaccine Effectiveness Against the Omicron (B.1.1.529) Variant in Hemodialysis Patients
Source: Kidney Int Rep. 2022 Apr 13;7(6):1406–9. doi: 10.1016/j.ekir.2022.04.005 (PMC9006399; doi:10.1016/j.ekir.2022.04.005)
Supplement: Supplementary File (PDF) [file mmc1.pdf]

SUPPLEMENTARY MATERIALS:

Contents

Supplementary Methods 1
Figure S1. Flow diagram of study cohort 3
Table S1. Clinical characteristics associated with Omicron infection in haemodialysis patients 4
Table S2. Clinical characteristics of patients receiving BNT1262b2 compared with ChAdOx1 5
Table S3. Clinical characteristics associated with Omicron infection 6
Table S4. Risk of SARS-CoV-2 infection by vaccination status and prior infection 7
Table S5. Treatments received by care setting 8
Table S6. Vaccine effectiveness against hospitalisation by vaccination status and prior infection 8
Supplementary References 9

Supplementary Methods

Patient Selection

One-thousand, one-hundred and twenty-one patients who were receiving haemodialysis within Imperial College Renal and Transplant Centre on the 1st December 2021, and were being prospectively studied as part of 'The Impact of COVID-19 on Patients with Renal disease and Immunosuppressed Patients' study were included. The study was approved by the Health Research Authority, Research Ethics Committee (Reference: 20/WA/0123). All, infection events were captured until 16th January 2022, with clinical follow up until 23rd January 2022. Clinical and vaccine data were obtained from electronic patient records and the institutional vaccine database, respectively.

Primary vaccine doses (2-doses for the purpose of this analysis), were either BNT162b2 or ChAdOx1, whilst all 3rd doses were of a mRNA based vaccine (BNT162b2), as per UK policy. The median time from 3rd vaccine to end of follow up was 91 (71-101) days compared with 286 (272-292) days from 2nd vaccine in those who only received 2-doses. Within the group who received 3 doses, the median time to infection post 3rd dose was 75 (53-93) days. There was no difference in follow up time between those who had infection post 3rd dose, 91 (67-106) days, compared with those without infection, 91 (71-100) days, p=0.36.

Detection of SARS-CoV-2 infection

All patients underwent weekly screening for asymptomatic infection by nasopharyngeal swabbing, with viral detection via reverse-transcriptase polymerase chain reaction (RT-PCR) assays. In addition, patients underwent additional screening for infection if symptomatic. The variant of interest, Omicron (B.1,1,529), was either detected by sequencing or latterly via the demonstration of S-gene drop out; infection was deemed of 'probable Omicron infection' if these criteria were not met, but overall percentage of sequenced cases within London was >96%. Reverse-transcriptase PCR was carried out using either the Thermofisher, Cepheid or Roche assays. Patients admitted to hospital for non-COVID related causes were screened twice a week for infection. Nosocomial transmission was defined as newly detected infection following a negative admission swab, in a patient who had contact (shared nursing bay), with a positive patient. If no positive contact, nosocomial infection would require an admission >7 days and 2 prior negative swabs.

SARS-CoV-2 antibody detection

Routine serological screening of haemodialysis patients every 3-months started in June 2020, as previously described[S1]. Serum was tested for antibodies to both the nucleocapsid protein (anti-NP) and spike protein (anti-S). Anti-NP was tested using the Abbott Architect SARS-CoV-2 IgG 2 step chemiluminescent immunoassay (CMIA) according to manufacturer's instructions. This is a non-quantitative assay and samples were interpreted as positive or negative with a threshold index value of 1.4. The presence of anti-NP was used as a marker of natural infection. For vaccine responses, anti-S IgG were assessed using the Abbott Architect SARS-CoV-2 IgG Quant II CMIA. Anti-S antibody titres are quantitative with a threshold value of 7.1 BAU/ml for positivity, and an upper level of detection of 5680 BAU/ml. Prior to December 2020, patients were initially screened for anti-NP, and those with a subthreshold anti-NP index value (0.25-1.4), underwent confirmatory testing for natural infection by assessing for receptor binding domain (anti-RBD) antibodies. This was performed using an in-house double binding antigen ELISA (Imperial Hybrid DABA; Imperial College London, London, UK), which detects total RBD antibodies

Definition of prior SARS-CoV-2 infection

Prior exposure was defined by a history of infection confirmed through viral detection from nasopharyngeal swab specimens, via reverse-transcriptase polymerase chain reaction (RT-PCR) assays, or by serological assessment.

Statistical Analysis

Statistical analysis was conducted using Prism 9.0 (GraphPad Software Inc., San Diego, California). Unless otherwise stated, all data are reported as median with interquartile range (IQR). The Chi-squared test was used for proportional assessments. The Mann-Whitney and Kruskal-Wallis tests were used to assess the difference between 2 or >2 groups, with Dunn's post-hoc test to compare individual groups.

Assessment of vaccine effectiveness

Reported outcomes included RT-PCR proven SARS-CoV-2 infection, hospitalisation, and death; death was recorded as SARS-CoV2 related if it occurred within 28 days of confirmed infection. Patients who died of non-SARS-CoV2 related causes, and those who received a transplant during follow up, were censored at the time of death or transplant respectively. Event rate for infection were reported as incidence per 1000-patient days at risk. Cox proportional hazards models were used to determine adjusted hazard ratios (HR) for the first PCR-positive test post 14 days after last vaccine. Patients were categorised by vaccination status on the 1st December, as unvaccinated, partially vaccinated (2 vaccines) or boosted (3 vaccines). Patients who received additional doses of a SAR-CoV-2 vaccine during the follow up period were censored at 14-days post inoculation. Only 6 patients received a 4th vaccine dose during the study period. Vaccine effectiveness (VE) was calculated using the formula VE= (1-adjusted HR) x100.

Figure S1. Flow diagram of study cohort.

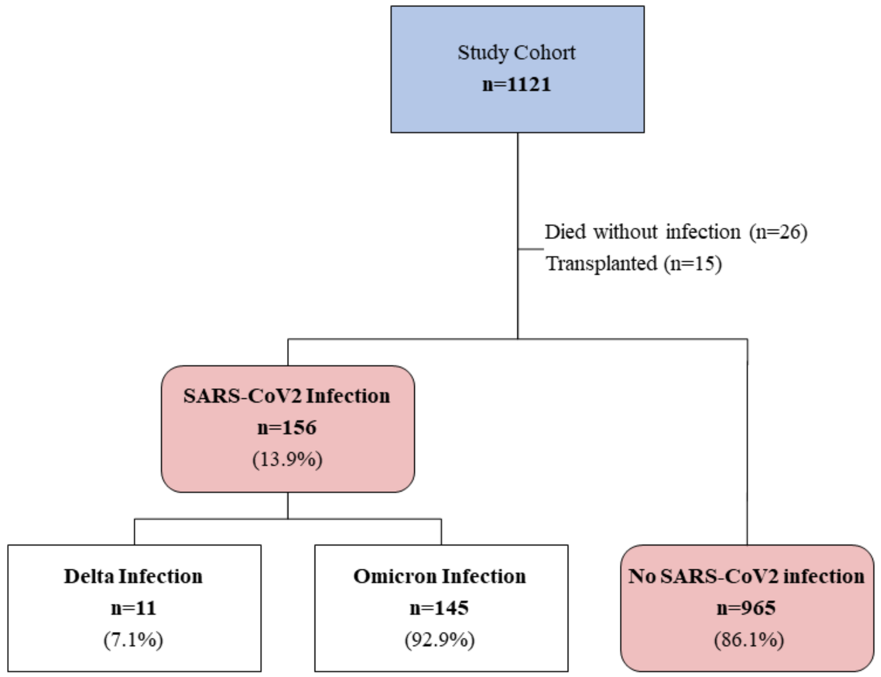

Table S1. Clinical characteristics associated with Omicron infection in haemodialysis patients.

Characteristics		Infection Free N=965 (%)	Omicron Infection N= 145 (%)	p value
**Gender**	Male<br>Female	595 (61.7)<br>370 (38.3)	84 (57.9)<br>61 (42.1)	0.39
**Age**	Years (Median)	66 (56-76)	61 (49-74)	**0.0035**
**Ethnicity**	Caucasian<br>Black*<br>Indoasian<br>Other	285 (29.5)<br>220 (22.8)<br>340 (35.2)<br>120 (12.4)	32 (22.1)<br>52 (35.9)<br>41 (28.3)<br>20 (13.8)	**0.0007**
**Cause of Kidney Failure**	Polycystic kidney disease<br>Glomerulonephritis*<br>Diabetic nephropathy<br>Urological<br>Unknown<br>Other	47 (4.9)<br>170 (17.6)<br>399 (41.3)<br>70 (7.3)<br>187 (19.4)<br>92 (9.5)	4 (2.8)<br>20 (13.8)<br>65 (44.8)<br>6 (4.1)<br>39 (26.9)<br>11 (7.6)	0.25
**Previous transplant**	Yes<br>No	190 (19.7)<br>775 (80.3)	24 (16.6)<br>121 (83.4)	0.37
**Immunosuppression at time of vaccine**	None<br>Yes	806 (83.5)<br>159 (16.5)	123 (84.8)<br>22 (15.2)	0.69
**Diabetes**	No<br>Yes	457 (47.4)<br>508 (52.6)	71 (49.0)<br>74 (51.0)	0.72
**Prior SARS-CoV2 infection**	No<br>Yes	449 (46.5)<br>516 (53.5)	82 (56.6)<br>63 (43.4)	**0.024**
**Vaccination status**	Unvaccinated<br>Partially vaccinated<br>Boosted	55 (5.7)<br>239 (24.8)<br>671 (69.5)	15 (10.3)<br>54 (37.2)<br>76 (52.4)	**0.0002**
**Vaccine type (2-doses)^**	BNT1262b2<br>ChAdOx1	495 (54.4)<br>415 (45.6)	56 (43.1)<br>74 (56.9)	**0.0156**

*Comparator. ^Vaccinated patients only

Table S2. Clinical characteristics of patients receiving BNT1262b2 compared with ChAdOx1

Characteristics		ChAdOx1<br>N=489 (%)	BNT1262b2<br>N=551 (%)	P value
**Gender**	Male	296 (60.5)	349 (63.3)	0.35
	Female	193 (39.5)	202 (36.7)	
**Age**	Years (Median)	65 (55-74)	66 (56-92)	0.10
**Ethnicity**	Caucasian	144 (29.4)	158 (28.7)	**0.04**
	Black*	124 (25.4)	110 (20.0)	
	Indoasian	163 (33.3)	207 (37.6)	
	Other	58 (11.9)	76 (13.8)	
**Cause of Kidney Failure**	Polycystic kidney disease	26 (5.3)	24 (4.4)	0.77
	Glomerulonephritis*	81 (16.6)	95 (17.2)	
	Diabetic nephropathy	197 (40.3)	241 (43.7)	
	Urological	35 (7.2)	39 (7.1)	
	Unknown	95 (19.4)	108 (19.6)	
	Other	55 (11.2)	44 (8.0)	
**Previous transplant**	Yes	103 (21.1)	98 (17.8)	0.18
	No	386 (78.9)	453 (82.2)	
**Immunosuppression at time of vaccine**	None	405 (82.8)	463 (84.0)	0.60
	Yes	84 (17.2)	88 (16.0)	
**Diabetes**	No	239 (48.9)	251 (45.6)	0.28
	Yes	250 (51.1)	300 (54.4)	
**Prior SARS-CoV2 infection**	No	263 (53.8)	291 (52.8)	0.75
	Yes	226 (46.2)	260 (47.2)	

Table S3. Clinical characteristics associated with Omicron infection

Variable	Reference Group	Unadjusted		Adjusted	
		HR (95% CI)	P value	HR (95% CI)	P value
Age	<65 years	1			
	≥65 years	0.63 (0.45-0.88)	0.007	0.74 (0.52-1.03)	0.075
Ethnicity	Black	1		1	
	Non-Black	0.54 (0.39-0.77)	0.0004	0.63 (0.44-0.90)	0.01
Immunosuppression	No	1			
	Yes	0.94 (0.58-1.45)	0.78	-	
Number of vaccines	Unvaccinated	1		1	
	Partially vaccinated	0.94 (0.54-1.72)	0.83	1.03 (0.60-1.91)	0.91
	Boosted	0.42 (0.25-0.77)	0.0024	0.50 (0.29-0.92)	0.0179
Prior infection	No	1		1	
	Yes	0.69 (0.50-0.96)	0.0289	0.63 (0.45-0.87)	0.0059

Table S4. Risk of SARS-CoV-2 infection by vaccination status and prior infection

Variable	Reference Group	HR (95% CI)	P value	Vaccine Efficacy
**Vaccine type**	Unvaccinated	1	-	
	Partially vaccinated-ChAdOx1	1.04 (0.57-1.97)	0.91	-
	Partially vaccinated -BNT162b2	0.83 (0.43-1.62)	0.57	-
	Boosted – ChAdOx1	0.53 (0.30-0.98)	0.034	47 (2-70)
	Boosted – BNT162b2	0.34 (0.19-0.64)	0.0005	66 (36-81)
**Vaccine plus prior infection**	Unvaccinated	1	-	
	Unvaccinated – prior infection	0.53 (0.18-1.47)	0.23	-
	Partially vaccinated	0.81 (0.39-1.82)	0.58	-
	Partially vaccinated – prior infection	0.62 (0.30-1.38)	0.20	-
	Boosted	0.39 (0.20-0.86)	0.01	61 (14-80)
	Boosted – prior infection	0.23 (0.11-0.52)	0.0001	77 (48-89)

Table S5. Treatments received by care setting

In-hospital treatment was dependent on clinical characteristics and treatment algorithms as per UK National Institute of Health and Care Excellence guidelines[S2]

	**Care Setting**	
	**Outpatient (N=128)**	**Inpatient (N=17)***
No directed therapy	76 (59.4%)	5 (29.4)[#]
Sotrovimab	46 (35.9%)	2 (11.8)
Molnupiravir	6 (4.7%)	
Dexamethasone		3 (17.6)[#]
Remdesivir		1 (5.9)
Dexamethasone plus Remdesivir		2 (11.8)
Sotrovimab plus Remdesivir		2 (11.8)[#]
Dexamethasone plus Remdesivir plus Sotrovimab		2 (11.8)[#]

*Including patients with nosocomial infection; [#]Patients who died

Table S6. Vaccine effectiveness against hospitalisation by vaccination status and prior infection

Variable	Reference Group	HR (95% CI)	P value	Vaccine Effectiveness
**Vaccination status**	Unvaccinated	1	-	
	Partially vaccinated	0.23 (0.03-1.91)	-	
	Boosted	0.40 (0.10-2.63)	-	
**Prior infection**	No	1	-	
	Yes	0.27 (0.06-0.89)	0.049	73 (11-94)

Supplementary References

S1. Clarke CL, Prendecki M, Dhutia A, et al. Longevity of SARS-CoV-2 immune responses in hemodialysis patients and protection against reinfection. *Kidney international* 2021; **99**(6): 1470-7.

S2. COVID-19 rapid guideline: managing COVID-19. https://www.nice.org.uk/guidance/ng191.
